# Direct and indirect costs of COPD progression and its comorbidities in a structured disease management program: results from the LQ-DMP study

**DOI:** 10.1186/s12931-019-1179-7

**Published:** 2019-10-10

**Authors:** Florian Kirsch, Anja Schramm, Larissa Schwarzkopf, Johanna I. Lutter, Boglárka Szentes, Manuel Huber, Reiner Leidl

**Affiliations:** 1 Institute of Health Economics and Health Care Management, Helmholtz Zentrum München, Neuherberg, Germany; 2 Munich School of Management and Munich Center of Health Sciences, Ludwig-Maximilians-Universität, München, Germany; 3 AOK Bayern, Service Center of Health Care Management, Regensburg, Germany; 4 German Center for Lung Research (DZL), Coprehensive Pneumology Center Munich (CPC-M), Hannover, Germany

**Keywords:** COPD, DMP, Healthcare utilization, Direct costs, Indirect costs

## Abstract

**Background:**

Evidence on the economic impact of chronic obstructive pulmonary disease (COPD) for third-party payers and society based on large real world datasets are still scarce. Therefore, the aim of this study was to estimate the economic impact of COPD severity and its comorbidities, stratified by GOLD grade, on direct and indirect costs for an unselected population enrolled in the structured German Disease Management Program (DMP) for COPD.

**Methods:**

All individuals enrolled in the DMP COPD were included in the analysis. Patients were only excluded if they were not insured or not enrolled in the DMP COPD the complete year before the last DMP documentation (at physician visit), had a missing forced expiratory volume in 1 s (FEV_1_) measurement or other missing values in covariates. The final dataset included 39,307 patients in GOLD grade 1 to 4. We used multiple generalized linear models to analyze the association of COPD severity with direct and indirect costs, while adjusting for sex, age, income, smoking status, body mass index, and comorbidities.

**Results:**

More severe COPD was significantly associated with higher healthcare utilization, work absence, and premature retirement. Adjusted annual costs for GOLD grade 1 to 4 amounted to €3809 [€3691–€3935], €4284 [€4176–€4394], €5548 [€5328–€5774], and €8309 [€7583-9065] for direct costs, and €11,784 [€11,257–€12,318], €12,985 [€12,531-13,443], €15,805 [€15,034–€16,584], and €19,402 [€17,853–€21,017] for indirect costs. Comorbidities had significant additional effects on direct and indirect costs with factors ranging from 1.19 (arthritis) to 1.51 (myocardial infarction) in direct and from 1.16 (myocardial infarction) to 1.27 (cancer) in indirect costs.

**Conclusion:**

The findings indicate that more severe GOLD grades in an unselected COPD population enrolled in a structured DMP are associated with tremendous additional direct and indirect costs, with comorbidities significantly increase costs. In direct cost category hospitalization and in indirect cost category premature retirement were the main cost driver. From a societal perspective prevention and interventions focusing on disease control, and slowing down disease progression and strengthening the ability to work would be beneficial in order to realize cost savings in COPD.

## Background

Chronic Obstructive Pulmonary Disease (COPD) is characterized by persistent respiratory symptoms and airflow limitation, which is slowly progressive and not fully reversible. There is no known cure for COPD, but the symptoms are treatable and disease progression can be delayed. The main risk factor for COPD is tobacco smoking but other environmental exposures such as biomass fuel exposure and air pollution may contribute [1]. COPD is projected to be the third leading cause of death by 2020, and globally the COPD burden is projected to increase in coming decades because of continued exposure to COPD risk factors and aging of the population [2].

For substantial share of COPD patients, COPD is associated with exacerbations, reduced or even insufficient lung function, and further concomitant chronic diseases such as cardiovascular diseases, skeletal muscle dysfunction, metabolic syndrome, osteoporosis, depression, anxiety, lung cancer and alcohol addiction thus contributing to overall disease severity and reducing health-related quality of life [1, 3–5].

Besides the disease burden, COPD is also associated with substantial economic costs. In the European Union, the total direct costs for COPD are estimated to be about 3% (€38.6 billion) of the total health care budget [6]. There is some evidence that the costs from a societal perspective of COPD are even greater, see the German study conducted by Wacker et al. [7]. Results indicate that, the estimated mean annual indirect excess costs, additional costs in patients with COPD compared to individuals without COPD, were between €8621 in GOLD grade 1 and €27,658 in GOLD grade 4, and exceeded the mean annual direct excess costs about 3 times in every GOLD grade.

From a decision makers perspective, there is a substantial need on detailed knowledge about disease severity related to health care expenditures and cost-driving effects in patients with COPD in a real world setting to provide a rational basis for investments and resource allocation in a health care system. Therefore, the aim of this study was to estimate the costs of COPD severity, stratified by GOLD grades, and its comorbidities, with a large real-world dataset from a statutory health insurance fund, for patients enrolled in the structured German Disease Management Program (DMP) for COPD.

## Methods

### Data

The analysis was based on pseudonymized claims data routinely documented for participants of the structured German DMP for COPD, offered by AOK Bayern, a large regional health insurance fund. In Germany, DMPs were introduced by a legal framework nationwide compatible with the solidarity principle including quality-of-care requirements, a strict accreditation process, and initially strong financial incentives for statutory health insurance funds to set up programs [8, 9]. Due to these strict requirements of the legislature, the DMPs offered by different statutory health insurance funds are only marginally different.

### Study population

All individuals enrolled in the DMP COPD (AOK curaplan) were included in the study. Persons were only excluded if they were not insured or not enrolled in the DMP COPD the complete year before the last DMP documentation (at physician visit), had a missing forced expiratory volume in 1 s (FEV_1_) measurement or other missing values or absurd measures in covariates e.g. height or weight. Enrollment in the DMP COPD ensures that patients had COPD, as diagnostic confirmation is necessary. Physicians were only allowed to enroll patients if the Tiffeneau-Index (FEV1/forced vital capacity (FVC)) was ≤70% and a) the reduction in FEV1 was < 80% of the target value or b) an increase in FEV1 by less than 15% and/or by less than 200 ml in 10 min after inhalation of a short-acting beta-2-sympathomimetic or 30 min after inhalation of a short-acting anticholinergic or c) an increase in FEV1 by less than 15% and/or by less than 200 ml after at least 14 days of systematic glucocorticosteroids or at least 28 days of inhaled glucocorticosteroids in a stable disease episode and evidence of airway resistance elevation or pulmonary hyperinflation or gas exchange disorder in patients with FEV1/FVC > 70% and a radiographic examination of the thoracic organs that has ruled out another symptom-explaining disease. For the grading of COPD GOLD grades in the patients we used percentage predicted values based on reference values from the Global Lung Function Initiative (grade 1: FEV_1_ ≥ 80%; grade 2: 50% ≥ FEV_1_ < 80%; grade 3: 30% ≥ FEV_1_ < 50%; grade 4: FEV_1_ < 30%) [10].

### Health care utilization and calculation of costs

Claims data include information on inpatient treatment, outpatient-physician treatment (general practitioner and medical specialist), prescribed medication, prescribed medical aids, prescribed remedies and rehabilitation. Furthermore absence from work with sickness notification is documented. Health care utilization was based on individual claims data for the year before the most recent DMP documentation with FEV_1_ measurement. The number of ambulatory physician contacts was based on the billing cases, each billing case included at least one contact date with the ambulatory system, which were divided into general practitioner, specialist and other physician (in case of missing physician group key in the claims data) visit. The number of hospital days was based on hospital invoices, and for medication usage the number of prescribed medications were summed up. Over-the-counter pharmaceuticals, non-pharmacy medicines, and dietary supplements were not included as they are not available in claims data.

Information on retirement/employment status was derived from the insurance status of the patients. Work absence in the last 12 months was based on summed up days with sickness notification handed into the statutory health insurance fund.

Overall health care expenditures included direct and indirect health care costs. Direct costs reflect costs out of the perspective of a third party payer and were calculated by summing up all cost categories in the last year. This included costs for hospitalization based on Diagnoses Related Groups (DRGs), outpatient care based on EBM (einheitlicher Bewertungsmaßstab), family doctor-centred health care (hausarztzentrierte Versorgung), and integrated care (integrierte Versorgung), medication based on pharmacy sales prices (including discount agreements), rehabilitation (if covered by AOK Bayern) based on rehabilitation invoices, aids and remedies based on invoices of health care supply stores, and travel expenses based on public transport tickets, invoices of taxis and a fixed fee per kilometer (by usage of private car). Lump-sum payments for physicians were considered in the period they were paid, even when the partially covered the period before or after. All direct costs were inflated to the year 2018, using the inflation rate as reported for Germany by the Organisation for Economic Co-operation and Development (OECD).

Indirect costs were considered only for patients under the age of 65 years and out of a societal perspective. Costs of sick days were calculated for participants with an insurance status, which indicated that the person was full- or part-time employed or self-employed. Costs per day of work loss were calculated by dividing 2018 German mean annual labor costs (€40,833) by 365 days, as sickness notification also include weekends and public holidays. Premature retirement before the regular retirement age of 65 years was based on insurance status and valued by mean annual German labor costs (€40,833).

In this analysis the human capital approach was preferred over the friction cost approach to calculate productivity losses in paid work for society, following current recommendations [11].

### Covariates and comorbidities

Patients’ characteristics, socio-economic variables, and data on comorbidities were assessed based on claims data and DMP documentation. Characteristics were patients’ age group, sex, smoking status, and body mass index (BMI, kg/m^2^). The socio-economic variable yearly income was based on the information the claims data provided for annual gross pay (Bruttojahresentgelt), retirement income, and pensions (Versorgungsbezüge). We considered the comorbidities diabetes, stroke, myocardial infarction, cancer and arthritis as they have been shown to be important in terms of prevalence and clinical relevance in COPD [7]. To identify the comorbidities ICD-10 code groups were used, which were mainly based on the Charlson-comorbidity index with small adjustments (see also Table 1). For each comorbidity a minimum of one inpatient or two secured outpatient diagnoses in two different quarters were required. This inclusion condition was derived from the German, morbidity-based risk adjustment scheme [12].

**Table 1:** ICD-10 codes comorbidities

Comorbidity	ICD-10 codes
Diabetes	E10.0, E10.1, E10.2, E10.3, E10.4, E10.5, E10.6, E10.7, E10.8, E10.9, E11.0, E11.1, E11.2, E11.3, E11.4, E11.5, E11.6, E11.7, E11.8, E11.9, E12.0, E12.1, E12.2, E12.3, E12.4, E12.5, E12.6, E12.7, E12.8, E12.9, E13.0, E13.1, E13.2, E13.3, E13.4, E13.5, E13.6, E13.7, E13.8, E13.9, E14.0, E14.1, E14.2, E14.3, E14.4, E14.5, E14.6, E14.7, E14.8, E14.9
Stroke	G45, G46, I60, I61, I62, I63, I64, I65, I66, I67, I68, I69, H34.0
Myocardial infarction	I21, I22, I25.2
Cancer	C00, C01, C02, C04, C04, C05, C06, C07, C08, C09, C10, C11, C12, C13, C14, C15, C16, C17, C18, C19, C20, C21, C22, C23, C24, C25, C26, C30, C31, C32, C33, C34, C37, C37, C39, C40, C41, C43, C45, C46, C47, C48, C49, C50, C51, C52, C53, C54, C55, C56, C57, C58, C60, C61, C62, C63, C64, C65, C66, C67, C68, C69, C70, C71, C72, C73, C74, C75, C76, C81, C82, C83, C84, C85, C88, C90, C91, C92, C93, C94, C95, C96, C97
Arthritis	M05, M06, M31.5, M32, M33, M35.1, M35.3, M36.0

### Statistical analysis

Characteristics of COPD patients across GOLD grades were compared using analysis of variance (ANOVA) for continuous variables and Chi^2^-tests for categorical variables. Unadjusted means of healthcare utilization and expenditures were compared between the four groups by non-parametric Kruskal-Wallis tests.

Generalized linear models (GLM) were conducted to quantify the association between GOLD in grades 1 to 4 and direct and indirect costs. As the distribution of costs is typically highly skewed, a gamma model with log-link was used [13, 14]. The exponents of the regression coefficients in the model can be interpreted as factors. In order to quantify the impact of comorbidities (smoking status, BMI, diabetes, stroke, myocardial infarction cancer, and arthritis), all analyses were performed with and without adjustment for comorbidities. This approach allowed division of the estimated excess costs of GOLD grades, additional costs caused by a more severe GOLD grade (2–4) compared to GOLD grade 1, into a part related to COPD severity itself and a part related to associated comorbidity. Interactions between comorbidity and GOLD stages were disregarded based on previous evidence on lacking interaction effects [7]. In the first step, the basic models included age group (< 55; 55–64; 65–74; > 74), sex, and income (<€5000; €5000- < €10,000; €10,000- < €15,000; €15,000- < €20,000; €20,000- < €30,000; €30,000- < €50,000; >€50,000) as covariates. Extended models considered additionally smoking status (current smoker; former smoker; never smoker), BMI group (underweight: BMI < 18.5; normal weight: BMI 18.5- < 25; overweight: BMI 25- < 30; obese: BMI ≥30), and the selected comorbidities (diabetes; stroke; myocardial infarction; cancer; arthritis). Recycled predictions were used to estimate absolute cost differences between GOLD grades based on the basis model and extended model. Separate cost analyses were conducted for each category of direct and indirect costs. However, for some of the categories, there were many observations with zero costs, which are not covered by the gamma distribution. Therefore, these models were estimated following a two-part approach [15, 16]: in the first step, the probability of incurring positive costs was modeled using a logistic model and, in the second step, the amount of positive costs was modeled for those who incurred positive costs, using a GLM with gamma distribution. One-part models were conducted for total direct, ambulatory, and medication costs, while two-part models were conducted for the direct cost categories hospitalization, rehabilitation, remedies and aid, and travel costs and for total indirect costs, sick days, and premature retirement. Confidence intervals and *p*-values for cost differences were derived by bootstrapping the original data set using 5000 replications [17]. The single cost categories in direct and indirect costs after bootstrapping not necessarily add up to the amount reported in direct and indirect costs.

All analyses were performed using the SAS statistical package version 9.4 (SAS Institute, Cary, NC, USA).

## Results

### Study population

**Fig. 1:**
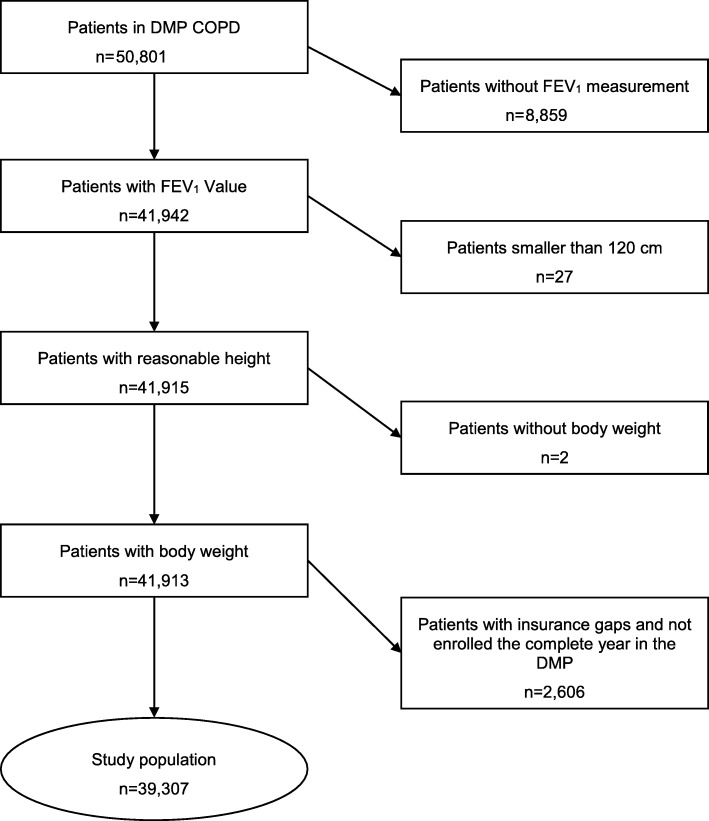
Patient selection

The selection of the study population is described in Fig. 1. The data set consisted of 50,801 patients who were enrolled in the DMP COPD. Of these, 8859 patients were excluded as no FEV_1_-value was available. Further, 27 patients were excluded as their height was below 1.20 m and for 2 patients the BMI could not be calculated due to missing body weight. Finally, 2606 patients were excluded as they were not insured or not enrolled in the DMP COPD the complete year before the last DMP documentation with FEV_1_ measurement. The final dataset contained 39,307 patients. Differences in patient characteristics (Additional file 1: Table S1) and in unadjusted health care utilization, work absenteeism and costs (Additional file 1: Table S2) can be found in the online appendix. Excluded patients were significantly younger with higher income, they were more often current smokers, and had lower prevalence in all comorbidities. Furthermore, patients excluded had significantly less physician contacts, were longer and more often hospitalized and had more medication prescriptions, in summary higher total direct health care expenditures. They were significantly more often employed, less often retired with a higher number of sick days, leading to overall lower total indirect costs.

Table 2 gives the characteristics of the study population by GOLD stage – with 76.8% of patients classified into GOLD grade 1 and 2. Direct comparison of the GOLD grades showed that the mean age ranged from 70.1 years in grade 2 to 67.8 years in grade 4 (*p* < 0.0001). The percentage of women decreased over GOLD grades from 52.4% in grade 1 to 32.9% in grade 4 (*p* < 0.0001). More patients in higher disease grades were in the lower income groups (*p* < 0.0001). The percentage of current smokers and former smokers was found to be higher in more severe grades (*p* < 0.0001). There was also a decline in BMI from 29.3 in grade 1 to 26.0 in grade 4 (*p* < 0.0001). The prevalence of considered comorbidities was more pronounced in the less severe GOLD grades 1 and 2 than in the more severe GOLD grades 3 and 4, except in case of myocardial infarction.

**Table 2:** Characteristics of the study population

	GOLD grade 1	GOLD grade 2	GOLD grade 3	GOLD grade 4	*p*-value
*N*	12,053 (30.7%)	18,119 (46.1%)	7383 (18.8%)	1752 (4.5%)	
Age (years)	70.0 (11.7)	70.1 (10.8)	70.0 (9.8)	67.8 (9.2)	< 0.0001
Age > 74 years	4900 (40.7%)	6980 (38.5%)	2587 (35.0%)	438 (25.0%)	< 0.0001
Age 65–74 years	3448 (28.6%)	5666 (31.3%)	2636 (35.7%)	682 (38.9%)	
Age 55–64 years	2443 (20.3%)	4016 (22.2%)	1733 (23.5%)	506 (28.9%)	
Age < 55	1262 (10.5%)	1457 (8.0%)	427 (5.8%)	126 (7.2%)	
Female	6319 (52.4%)	8268 (45.6%)	2701 (36.6%)	577 (32.9%)	< 0.0001
income < €5.000	2915 (24.2%)	4222 (23.3%)	1600 (21.7%)	382 (21.8%)	< 0.0001
income€5000 < €10,000	3920 (32.5%)	6140 (33.9%)	2740 (37.1%)	713 (40.7%)	
income €10,000 < €15,000	2486 (20.6%)	3868 (21.4%)	1595 (21.6%)	333 (19.0%)	
income €15,000 < €20,000	944 (7.8%)	1416 (7.8%)	544 (7.4%)	113 (6.5%)	
income €20,000 < €30,000	1020 (8.5%)	1383 (7.6%)	497 (6.7%)	112 (6.4%)	
income €30,000 < €50,000	669 (5.6%)	936 (5.2%)	360 (4.9%)	76 (4.3%)	
income ≥ €50.000	99 (0.8%)	154 (0.9%)	47 (0.6%)	23 (1.3%)	
Current smokers	3143 (26.1%)	5822 (32.1%)	2631 (35.6%)	600 (34.3%)	< 0.0001
Former smokers (quit within the last 8 years)	1343 (11.1%)	2451 (13.5%)	1327 (18.0%)	402 (23.0%)	
Never smokers or former smokers (quit > 8 years ago)	7567 (62.8%)	9846 (54.3%)	3425 (46.4%)	750 (42.8%)	
BMI (kg/m^2^)	29.3 (5.8)	29.3 (6.2)	27.9 (6.2)	26.1 (6.1)	< 0.0001
Normal weight (18.5 ≤ BMI < 25)	2578 (21.4%)	4254 (23.5%)	2243 (30.4%)	681 (38.9%)	< 0.0001
Overweight (25 ≤ BMI < 30)	4523 (37.5%)	6370 (35.2%)	2449 (33.2%)	517 (29.5%)	
Obese (BMI ≥ 30)	4834 (40.1%)	7247 (40.0%)	2397 (32.5%)	408 (23.3%)	
Underweight (BMI < 18.5)	118 (1.0%)	248 (14.4%)	294 (4.00%)	146 (8.3%)	
Diabetes	4306 (35.7%)	6669 (36.8%)	2574 (34.9%)	481 (27.5%)	< 0.0001
Stroke	3509 (29.1%)	5444 (30.1%)	2055 (27.8%)	406 (23.2%)	< 0.0001
Myocardial infarction	1430 (11.9%)	2371 (13.1%)	1069 (14.5%)	244 (13.9%)	< 0.0001
Cancer	3612 (30.00%)	5708 (31.5%)	2318 (31.4%)	470 (26.8%)	< 0.0001
Arthritis	1853 (15.4%)	2462 (13.6%)	824 (11.2%)	145 (8.3%)	< 0.0001

### Health care utilization, work absence and retirement

Table 3 shows unadjusted frequencies of health care utilization, work absence, and retirement. Overall, 100% in every GOLD grade had a physician contact in the last 12 months. The mean absolute visits ranged between 14.1, 7.8, and 5.3 in grade 4 (lowest) to 16.0, 8.4, and 6.4 in grade 2 (highest) for total physician, general practitioner and specialist visits, respectively. The number of patients with at least one hospital stay and the number of total hospital days in the last 12 months increased with a factor of 2 from grade 1 to grade 4. Even the number of patients who received a prescription for a medication varied less than 2% (97.61 in grade 1 to 99.14% in grade 4), and the absolute number of prescribed medications was more than 40% higher in grade 4 compared to grade 1. The percentage of persons in working age prematurely retired was more than twice as high, and the percentage of persons employed was less than half in grade 4 compared to grade 1. While the number of sick days, in persons with reported sick days, increased over 50% with disease severity.

**Table 3:** Unadjusted healthcare utilization, work absenteeism and resulting costs

	GOLD grade 1	GOLD grade 2	GOLD grade 3	GOLD grade 4	*p*-value
*N*	12,053 (30.7%)	18,119 (46.1%)	7383 (18.8%)	1752 (4.5%)	
Healthcare utilization					
Outpatient services					
% User	100.0	100.0	100.0	100.0	1.0000
Total number of visits	15.8 (8.7)	16.0 (9.0)	15.3 (8.7)	14.1 (8.0)	< 0.0001
General practitioner	8.2 (5.7)	8.4 (5.9)	8.2 (5.8)	7.8 (5.5)	< 0.0001
Specialist	6.3 (5.4)	6.4 (5.6)	6.0 (5.3)	5.3 (4.6)	< 0..0001
Inpatient services					
% User	10.9	12.7	17.5	23.0	< 0.0001
Number of hospital days	4.3 (12.5)	4.8 (12.6)	6.6 (14.6)	8.9 (17.2)	< 0.0001
Prescribed medication					
%User	97.6	98.3	98.8	99.1	< 0.0001
Number of prescribed drugs	15.4 (12.1)	17.0 (12.6)	20.0 (14.5)	22.1 (14.8)	< 0.0001
Direct costs					
Outpatient costs	€548	€600	€604	€564	0.0109
Inpatients costs	€2034	€2271	€3042	€4192	< 0.0001
Medication costs	€593	€716	€932	€1478	< 0.0001
Rehabilitation	€130	€146	€176	€232	< 0.0001
Aids and Remedies	€404	€428	€603	€1016	< 0.0001
Travel costs	€112	€131	€203	€320	< 0.0001
Total direct costs	€3821	€4292	€5561	€7801	< 0.0001
	GOLD grade 1	GOLD grade 2	GOLD grade 3	GOLD grade 4	p-value
*N*	3705 (31.0%)	5473 (45.7%)	2160 (18.1%)	632 (5.3%)	
Works absenteeism (participants < 65 years)					
%retired	22.0	25.6	37.2	50.6	< 0.0001
%employed	52.9	47.0	38.3	25.6	< 0.0001
% with sick days	78.8	80.0	81.2	81.5	0.4724
Number of sick days	43.3	44.9	53.0	66.1	0.0001
Indirect costs (participants < 65 years)					
Sick days	€3069	€3148	€3138	€2737	< 0.0001
Premature retirement	€8777	€10,188	€14,743	€20,195	< 0.0001
Total indirect costs	€11,846	€13,336	€17,880	€22,932	< 0.0001

### Costs

In Table 3 unadjusted direct and indirect costs are shown. Unadjusted annual direct health care costs were increased by factors 1.12 in grade 2, 1.46 in grade 3, and 2.04 in grade 4 compared to grade 1. Besides outpatient costs, all cost categories increased from grade 1 to grade 4 with factors ranged from 1.78 for rehabilitation costs to 2.86 for travel costs. Indirect costs increased with disease severity and were almost twice as high in grade 4 compared to grade 1. This difference arose completely from costs for premature retirement, as mean costs for sick days were lowest in grade 4.

Table 4 shows the results of the regression analyses for costs. Adjusted factors of excess costs of GOLD grades 2 to 4 ranged from 1.13 (1.10–1.16) to 2.18 (2.06–2.32) in the basic model and from 1.12 (1.09–1.15) to 2.29 (2.16–2.43) in the extended model for direct costs and from 1.08 (0.99–1.18) to 1.64 (1.37–1.96) in the basic model and 1.08 (0.99–1.18) to 1.74 (1.45–2.09) in the extended model for indirect costs.

**Table 4:** Effect of COPD on annual direct and indirect costs – basic and extended models

Covariate		Direct costs		Indirect cots	
		Basic model	Extended model	Basic model	Extended model
		Exp(beta) (CI 95%)	Exp(beta) (CI 95%)	Exp(beta) (CI 95%)	Exp(beta) (CI 95%)
Intercept		2349.13 (2236.80-2467.10)	2047.55 (1937.01-2164.19)	**4225.11 (3770.26-4734.82)**	3425.83 (2959.28-3965.95)
GOLD	Grade 1	1.00	1.00	**1.00**	1.00
	Grade 2	**1.13 (1.10–1.16)**	**1.12 (1.09–1.15)**	**1.08 (0.99–1.18)**	**1.08 (0.99–1.18)**
	Grade 3	**1.46 (1.41–1.51)**	**1.45 (1.40–1.50)**	**1.33 (1.19–1.49)**	**1.36 (1.21–1.52)**
	Grade 4	**2.18 (2.06–2.32)**	**2.29 (2.16–2.43)**	**1.64 (1.37–1.96)**	**1.74 (1.45–2.09)**
Age (years)	< 55	1.00	1.00	1.00	1.00
	55–64	**1.34 (1.28–1.40)**	**1.17 (1.12–1.23)**	**1.96 (1.80–2.14)**	**1.85 (1.70–2.02)**
	65–74	**1.57 (1.50–1.64)**	**1.26 (1.20–1.32)**	–	–
	> 74	**1.81 (1.73–1.89)**	**1.39 (1.32–1.46)**	–	–
Sex	Female	1.00	1.00	1.00	1.00
	Male	**1.04 (1.02–1.07)**	1.00 (0.97–1.02)	1.03 (0.95–1.12)	1.03 (0.95–1.12)
Income	% income < €5.000	1.00	1.00	1.00	1.00
	% income€5000 < €10,000	**1.07 (1.04–1.10)**	**1.05 (1.02–1.08)**	**3.20 (2.85–3.59)**	**3.18 (2.83–3.57)**
	% income €10,000 < €15,000	**1.06 (1.02–1.10)**	**1.04 (1.01–1.08)**	**2.14 (1.87–2.46)**	**2.14 (1.87–2.45)**
	% income €15,000 < €20,000	0.97 (0.93–1.02)	0.99 (0.94–1.03)	**1.46 (1.26–1.68)**	**1.49 (1.29–1.38)**
	% income €20,000 < €30,000	**0.81 (0.77–0.87)**	**0.85 (0.81–0.89)**	**1.17 (1.03–1.31)**	**1.23 (1.09–1.38)**
	% income €30,000 < €50,000	**0.82 (0.77–0.87)**	**0.87 (0.82–0.92)**	1.00 (0.89–1.14)	1.05 (0.93–1.19)
	% income ≥ €50.000	**0.84 (0.74–0.96)**	0.88 (0.78–1.00)	1.01 (0.78–1.32)	1.04 (0.80–1.35)
Smoking status	Never smokers or former smokers (quit > 8 years ago)		**1.00**		1.00
	Current smokers		**0.91 (0.89–0.94)**		0.94 (0.86–1.03)
	Former smokers (quit within the last 8 years)		**1.12 (1.08–1.16)**		1.03 (0.91–1.15)
Weight	Normal weight		1.00		1.00
	Underweight		**1.29 (1.18–1.40)**		1.24 (0.97–1.57)
	Overweight		**0.92 (0.89–0.95)**		1.02 (0.92–1.13)
	Obese		0.99 (0.96–1.02)		**1.16 (1.06–1.27)**
Comorbidities	Diabetes		**1.32 (1.29–1.36)**		**1.22 (1.11–1.35)**
	Stroke		**1.30 (1.27–1.34)**		**1.25 (1.13–1.39)**
	Infarction		**1.51 (1.46–1.57)**		**1.16 (1.01–1.34)**
	Cancer		**1.38 (1.35–1.42)**		**1.27 (1.16–1.39)**
	Arthritis		**1.19 (1.15–1.23)**		**1.22 (1.07–1.39)**

bold: *p* > 0.05

In the extended model the effects of GOLD grades hardly changed or even increased slightly, compared to the basic model, as shown in Table 4. Compared to never smokers, active smoking tend to decrease costs with a factor of 0.91 (0.89–0.94) and 0.94 (0.86–1.03), while former smokers induce higher costs with a factor of 1.12 (1.08–1.16) and 1.03 (0.91–1.15) for direct and indirect costs, respectively. Compared to normal weight, underweight induce higher direct and indirect costs with a factor of 1.29 (1.18–1.40) and 1.24 (0.97–1.57), respectively, while overweight and obesity tend to reduce indirect costs with a factor of 0.92 (0.89–0.95) and 0.99 (0.96–1.02), and increase indirect costs with a factor of 1.02 (0.92–1.13) and 1.16 (1.01–1.34) respectively. The presence of additional diseases had significant additional effects on direct and indirect costs, these effects were more pronounced in direct than in indirect costs and increased costs with a factor ranged from 1.16 (1.01–1.39) for myocardial infarction in indirect costs to 1.51 (1.46–1.57) for myocardial infarction in direct costs.

Adjusted mean annual costs for GOLD grades 1 to 4 from the basic model are illustrated in Table 5. Corresponding direct costs were €3809 [€3691–€3935] in grade 1, €4284 [€4176–€4394] in grade 2, €5548 [€5328–€5774] in grade 3, and €8309 [€7583–€9065] in grade 4. Indirect costs amounted to €11,784 [€11,257–€12,318] in grade 1, €12,985 [€12,531–€13,443] in grade 2, €15,805 [€15,034–€16,584] in grade 3, and €19,402 [€17,853–€21,017] in grade 4. Similar results were estimated in the extended model, which are presented in Table 6.

**Table 5:** Adjusted mean annual direct and indirect costs of GOLD Grades – Basic model

	GOLD grade 1		GOLD grade 2		GOLD grade 3		GOLD grade 4	
	*N*	Mean (CI 95%)	*N*	Mean (CI 95%)	*N*	Mean (CI 95%)	*N*	Mean (CI 95%)
Total direct costs	12,053 (30.7%)	€3809 [€3691–€3935]	18,119 (46.1%)	€4284 [€4176–€4394]***	7383 (18.8%)	€5548 [€5328–€5774]***	1752 (4.5%)	€8309 [€7583–€9065]***
Ambulatory costs	12,053 (30.7%)	€553 [€533–€574]	18,119 (46.1%)	€601 [€579–€623]***	7383 (18.8%)	€588 [€558–€621]	1752 (4.5%)	€579 [€511–€662]
Medication costs	12,053 (30.7%)	€594 [€571–€619]	18,119 (46.1%)	€716 [€688–€746]***	7383 (18.8%)	€930 [€877–€994]***	1752 (4.5%)	€1486 [€1176–€1862]***
Hospitalization	12,053 (30.7%)	€2045 [€1946–€2147]	18,119 (46.1%)	€2268 [€2184–€2354]***	7383 (18.8%)	€3000 [€2836–€3169]***	1752 (4.5%)	€4272 [€3819–€4711]***
Rehabilitation costs	12,053 (30.7%)	€128 [€116–€141]	18,119 (46.1%)	€145 [€134–€157]*	7383 (18.8%)	€179 [€160–€201]***	1752 (4.5%)	€259 [€215–€305]***
Remedies	12,053 (30.7%)	€400 [€384–€417]	18,119 (46.1%)	€427 [€413–€442]*	7383 (18.8%)	€609 [€579–€640]***	1752 (4.5%)	€1068 [€978–€1158]***
Travel costs	12,053 (30.7%)	€113 [€105–€122]	18,119 (46.1%)	€130 [€186–€214]**	7383 (18.8%)	€200 [€186–€214]***	1752 (4.5%)	€321 [€279–€366]***
Total indirect costs	3705 (31.0%)	€11,784 [€11,257–€12,318]	5473 (45.7%)	€12,985 [€12,531–€13,443]**	2160 (18.1%)	€15,805 [€15,034–€16,584]***	632 (5.3%)	€19,402 [€17,853–€21,017]***
Sick day costs	3705 (31.0%)	€2941 [€2750–€3132]	5473 (45.7%)	€3162 [€2984–€3344]	2160 (18.1%)	€3256 [€2975–€3534]	632 (5.3%)	€3195 [€2633–€3806]
Premature retirement costs	3705 (31.0%)	€9105 [€8622–€9575]	5473 (45.7%)	€9762 [€9361–€10,159]*	2160 (18.1%)	€12,247 [€11,582–€12,919]***	632 (5.3%)	€15,634 [€14,390–€16,901]***

Significant differences between GOLD grade 1 and the other groups:* *p* < 0.05 ** *p* < 0.01 *** *p* < 0.001.

Confidence intervals and *p*-values of the cost differences were derived by bootstrapping the original data set with 5000 bootstraps [17]

bold: *p* > 0.05

**Table 6:** Adjusted mean annual direct and indirect costs of GOLD Grades – Extended model

	GOLD grade 1		GOLD grade 2		GOLD grade 3		GOLD grade 4	
	*N*	Mean (CI 95%)	*N*	Mean (CI 95%)	*N*	Mean (CI 95%)	*N*	Mean (CI 95%)
Total direct costs	12,053 (30.7%)	€3828 [€3706–€3954]	18,119 (46.1%)	€4295 [€4185–€4407]***	7383 (18.8%)	€5540 [€5323–€5769]***	1752 (4.5%)	€8779 [€7982–€9649]***
Ambulatory costs	12,053 (30.7%)	€554 [€533–€577]	18,119 (46.1%)	€597 [€575–€619]**	7383 (18.8%)	€595 [€566–€626]*	1752 (4.5%)	€615 [€546–€697]
Medication costs	12,053 (30.7%)	€590 [€568–€613]	18,119 (46.1%)	€718 [€690–€748]***	7383 (18.8%)	€939 [€888–€1001]***	1752 (4.5%)	€1498 [€1208–€1852]***
Hospitalization	12,053 (30.7%)	€2068 [€1969–€2170]	18,119 (46.1%)	€2271 [€2189–€2355]**	7383 (18.8%)	€2943 [€2784–€3112]***	1752 (4.5%)	€4316 [€3850–€4781]***
Rehabilitation costs	12,053 (30.7%)	€126 [€113–€138]	18,119 (46.1%)	€145 [€134–€156]*	7383 (18.8%)	€183 [€163–€205]***	1752 (4.5%)	€275 [€226–€325]***
Remedies	12,053 (30.7%)	€394 [€378–€411]	18,119 (46.1%)	**€421** [€407–€435]*	7383 (18.8%)	€632 [€601–€664]***	1752 (4.5%)	€1185 [€1086–€1287]***
Travel costs	12,053 (30.7%)	€114 [€106–€123]	18,119 (46.1%)	€131 [€123–€138]**	7383 (18.8%)	€196 [€183–€210]***	1752 (4.5%)	€319 [€278–€361]***
Total indirect costs	3705 (31.0%)	€11,739 [€11,208-12,280]	5473 (45.7%)	€12,984 [€12,523–€13,443]***	2160 (18.1%)	€16,117 [€15,312–€16,922]***	632 (5.3%)	€20,525 [€18,822–€22,287]***
Sick day costs	3705 (31.0%)	€2983 [€2789–€3183]	5473 (45.7%)	€3145 [€2968-33,329€]	2160 (18.1%)	€3210 [€2924–€3496]	632 (5.3%)	€3255 [€2668–€3900]
Premature retirement costs	3705 (31.0%)	€8972 [€8497–€9449]	5473 (45.7%)	€9715 [€9315–€10,108]*	2160 (18.1%)	€12,449 [€11,792–€13,130]***	632 (5.3%)	€16.180 [€14,936–€17.443]***

Significant differences between GOLD grade 1 and the other groups:* *p* < 0.05 ** *p* < 0.01 *** *p* < 0.001.

Confidence intervals and *p*-values of the cost differences were derived by bootstrapping the original data set with 5000 bootstraps [17]

bold: *p* > 0.05

## Discussion

### Main results

This study estimated overall health care expenditures of unselected COPD patients enrolled in a structured German DMP for COPD in different severity grades using real world data. Progression of COPD is associated with tremendous costs for third party payers, but even more for society as indirect costs exceed direct costs almost 2 times in grade 3 and 4 and more than 3 times in grade 1 and 2. Adjusting for major comorbidities did not decrease the effect of more pronounced disease severity of COPD on costs, so that comorbidities independently added to the direct as well as indirect costs.

### Comparison of findings with literature

When comparing our results to previously published work, we observe a huge variance of reported costs for COPD.

A systematic review [18] of 7 COPD studies in Germany found annual costs per COPD patient between €1212 and €3492 (price year 2010) from a societal perspective. Most studies are limited to singular direct [4, 19, 20] or indirect [21] cost categories. Only three [22–24] studies estimated direct and indirect costs, which were restricted to mean annual costs per COPD patient and did not report stratified costs for GOLD grades. Further, the inclusion and exclusion criteria for the study population differed from our approach. It seemed that Weißflog et al. [24] had the methodological approach most similar to ours, they identified COPD patients with an ICD-9 code of 493 in claims data of a statutory health insurance fund. Resulted in mean annual costs for COPD patients of €3492 although, compared to our analysis, in direct costs ambulatory, remedy and aid, and travel costs were missing and in indirect costs additionally premature death were included. Besides different methods and data sources of the studies considered in the review, it seems there is an increase of costs over time in the direct cost categories.

A similar trend of increasing costs per patient for COPD could be found in most international studies. Dal Negro [25] found mean direct and indirect costs, in different populations, of €1801 in 2002, €2724 in 2008, and €3291 in 2015, which is an total in increase of 82.7% within 13 years in Italy. For Korea Lim et al. [26] found in patients with mild to moderate COPD direct costs of US$264 in 2007 and US$797 in 2012 in the same population, which is an increase of 301.9%. Further, Kim et al. [27] measured for Korea an increase in in- and outpatient care from US$2217 in 2006 to US$2802 in 2010 in different populations, which is an increase of 25.3%. Jansson et al. [28] estimated an uninflated increase from 1999 to 2010 in total direct and indirect costs in GOLD grade 1 from SEK 2513 to SEK 5686 (226.3%), in grade 2 from SEK 8277 to SEK 30,957 (374.0%), in grade 3 from SEK 42,856 to SEK 54,242 (126.6%), and in grade 4 from SEK 107,992 to SEK 165,569 (53.3%) in a different study populations in Sweden. For the US Blanchette et al. [29] found an increase in direct costs from US$11,807 in 1987 to US$16,135 in 2007 in different study populations, which is an increase of 38%. Dalal et al. [30] estimated an increase in direct costs from US$4.006 in 2006 to US$5168 in 2009 in commercially insured and from US$4138 in 2006 to US$4659 in 2009 in Medicare COPD patients, which is an increase of 29.0 and 12.6% respectively in varying cohorts. For Germany Byng et al. [31] found an increase in inflation-adjusted mean annual costs from €6739 to €7091 within 18 months in the same cohort of COPD patients, which is approximately 5%.

Contrarily Tsai et al. [32] found a 12.4% decrease of direct costs from US$3434 in 2004 to US$3008 in 2010 in Taiwan, mainly attributable to decreased average numbers of hospital and intensive care unit admissions. Also for the US Nurmagambetov et al. [33] measured direct costs for COPD in a working age patients and found a decline in expenditures from US$1400 in 1999 to US$960 in 2003 in varying populations, which is mainly attributable to a reduction in hospital admissions.

The most recently published German study from Wacker et al. [7] calculated annual direct and indirect cost of COPD based on data from the German COPD cohort COSYCONET (German **CO**PD and **Sy**stematic Consequences – **Co**morbidities **Net**work). COSYCONET is a prospective, observational, multicenter study that included 2741 patients aged 40 years or older with physician-diagnosed COPD. The LQ-DMP study also includes patients younger than 40 years, but only 130 individuals (0.33%) were aged younger than 40. Thus we think that including the entire range does not crucially affect comparability. The COSYCONET study might be prone to a specific selection of patients, as patients with a previous lung transplantation or lung volume reduction surgery and lung malignancies were excluded. Furthermore, only clinical stable patients, defined as having no moderate or severe exacerbations in the last four weeks before enrollment were included. Besides that, patients had to visit a study center for several hours for medical examination and data collection, which might exclude patients in bad overall conditions. This might lead to overall more healthy patients in each GOLD grade compared to the LQ-DMP study.

All in all, the COSYCONET cohort is on average more than 4 years younger and the BMI is two points lower. The prevalence of comorbidities was higher in the LQ-DMP cohort for all considered diseases, for diabetes, stroke myocardial infarction, cancer and arthritis by 22.6, 24.6, 4.1, 19.9, and 5.13%, respectively. The much higher prevalence of never smokers 54.9% vs. 5.4% and the much lower prevalence of former smokers 68.1% vs. 14.1% in LQ-DMP is due to the fact that we only had data on smoking status for the last 8 years, and if patients haven’t smoked during that period we classified them as never smokers. Despite these differences the factors regarding direct and indirect costs for current smokers of 0.93 (0.79–1.09) and 0.96 (0.68–1.36) in Wacker et al. and 0.91 (0.89–0.94) and 0.94 (0.86–1.03) in our analysis and for former smokers and 1.04 (0–91-1.18) and 1.24 (0.93–1.67) in Wacker et al. and 1.12 (1.08–1.16) and 1.03 (0.91–1.15) in our analysis are quite similar. The reduced costs for smokers and the higher costs for former smokers might be prone to reverse causation, since former smokers might quit smoking because of a recent adverse event (e.g. myocardial infarction or severe exacerbations).

The reported annual direct costs in COSYCONET were higher than in LQ-DMP by a factor of 1.17, 1.24, 1.41, and 1.29 for GOLD grade 1, 2, 3, and 4, respectively. Similarly, higher costs arise in COSYCONET for annual indirect costs. The factors were of 1.25 in grade 1, 1.23 in grade 2, 1.44 in grade 3, and 1.74 in grade 4 and exceed the factors for direct costs. The higher reported direct costs in COSYCONET might be caused by the use of a weighted average costs catalogue [34] to estimate costs from health care utilization. It seems that the catalogue does not reflect real health care expenditures adequately, as health utilization in COSYCONET and LQ-DMP are more similar than the cost estimates. This argument is further supported by the fact that in LQ-DMP additionally the costs for travel, rehabilitation and remedy and aid were considered, which are missing in COSYCONET. On the other hand, the indirect cost estimations in LQ-DMP might be less realistic as not all sickness notifications are handed into the statutory health care insurance fund and calculations on costs for premature retirement on basis of insurance status might be not as precise as on asking the patients directly as in COSYCONET.

Further, both studies conducted models estimating the effect of COPD GOLD grade in the basic model, and GOLD grade plus additional comorbidities in the extended model, on direct and indirect costs. The only difference in covariates was that Wacker et al. [7] used education and we used annual income as proxy for socioeconomic status. All in all, the results are again fairly similar, as there is a major overlap in direction and significance of factor estimates. Main differences are slightly higher factor estimates in COSYCONET for COPD GOLD grades which might be caused by the overall higher estimated costs found for COPD. Further, the influence of all comorbidities in both studies on direct costs is significant, although the factors again are slightly higher in the study conducted by Wacker et al. [7] ranging from 1.19 for diabetes to 1.77 for cancer, compared to ours ranging from 1.19 for arthritis to 1.51 for myocardial infarction. The higher factors for single comorbidities in COSYCONET might be the reason of the overall lower prevalence of comorbidities, which might be caused by the different methods (self-reported vs. two ambulatory diagnoses in two different quarters or one stationary diagnosis) of measuring comorbidities. Contrarily, for indirect costs the factors of comorbidities range from 1.08 for diabetes to 1.46 for stroke in Wacker et al. [7] and from 1.16 for myocardial infarction to 1.27 for cancer in our analysis, but only the influence in our study is significant, which might be caused by the larger population considered in our study.

In a review and three recently published articles the influence of different comorbidities on costs in COPD patients were considered. The review conducted by Huber et al. [35] estimated the mark-up on costs caused by several comorbidities in patients with COPD. They found a mark-up on total direct costs of 1.29 [36] for hypertension, 1.35 [36] for coronary heart failure, 1.36 [36] for diabetes without complications, 1.42 [36] for AIDS, 1.73 [36] for liver disease, 1.88 [37] for sleep apnea, 1.99 [36] for peptic ulcer, 2.10 [38] anemia, 2.18 [36] diabetes with complications, 2.00 [39], 2.24 [40], and 2.54 [41] for pneumonia, and 1.68 [42] and 2.83 [43] for cardiovascular disease. Mannino [44] et al. found odds ratios for total direct costs of 1.15 for asthma, 1.18 for osteoporosis, 1.23 for diabetes, 1.35 for depression, 1.43 for chronic kidney disease, 1.55 for cardiovascular disease, and 1.54 for anemia. Wacker et al. [45] considered the influence of 16 different comorbidities in COPD patients on direct costs and found a factor of 1.14 for peripheral artery disease, 1.15 for Migraine, 1.19 for cholecystitis/gallstones, 1.20 gastric ulcer, 1.21 for sleep apnea, 1.23 for cancer, 1.25 for heart disease, 1.36 for psychiatric disorders, and 1.38 for osteoporosis on direct costs. Chen et al. [46] found excess costs in COPD patients of €137 for infectious disease, €176 psychiatric disorders, €193 digestive disorders, €220 respiratory disorders other than COPD, and €491 circulatory disease. These previous studies considered different comorbid conditions at different aggregation levels, which hampers a straightforward comparison with our findings. The most frequently included comorbidity in previous publications is diabetes, and our factor of 1.32 is within the range of published estimates (1.23 [44] to 2.18 [36]). Furthermore, we estimated a factor of 1.51 for myocardial infarction which is also within the range of other circulatory disorders reported ranging from 1.25 [45] for heart disease [36] to 2.83 [43] for cardiovascular disease. For cancer we found a factor of 1.38 on direct costs which is higher than in the literature where factors were found to range between 1.23 [45] and 1.31 [7]. Results regarding stroke and arthritis could not be found in the literature besides those reported by Wacker et al. [7]. The available evidence is heterogeneous and far from comprehensive, but we still might overestimate the influence of the considered comorbidities and COPD itself on costs by not considering relevant comorbidities.

### Limitations

Our analysis is also subject to several limitations. The results are based on claims data of a large single statutory health insurance fund (AOK Bayern). Therefore the transferability of the findings might be limited, and claims data are collected for administrative and billing rather than for research purposes, and as characteristics of the insured can differ across statutory health insurance funds. Further, the population consists of patients enrolled in the DMP COPD which is voluntary that could cause self-selection effects, for example, that persons who are more health-conscious or more active overall would more often decide to take part in a DMP. While patients who are not adherent to the requirements of the DMP (continuous physician care), which might be predominantly patients in more severe GOLD grades, are excluded from the DMP. Further, physicians might enroll patients with special characteristics with a higher likelihood in a DMP [47]. Therefore, our population might be prone to an underestimation of health care provision and costs. On the other hand, we used for our analysis a completely unselected cohort of patients enrolled in the DMP COPD, as the only exclusion criteria were gap in insurance and DMP enrollment and missing values in covariates. Therefore, the estimated health care expenditures should reflect reality quite good.

The determination of the contact frequency with physicians in claims data is associated with methodological limitations. In recent year’s physician reimbursement was strongly shifted from fee-for service to lump-sum that means more contacts might be covered by one lump-sum fee, which tend to lead to an underestimation of the contact incidence in our study. Despite the presumption, the contact frequencies are quite in line with the rates reported by Wacker et al. [7].

The rather restrictive inclusion of comorbidities in the statistical analyzes may have led to an overestimation of the estimates of the COPD severity grades as well as the included comorbidities on costs, but it also limits the problem of multicollinearity between comorbidities, that affects individual predictors negatively. Further, prevalence of comorbidities might be overestimated in claims data, as there are financial incentives for hospitals for up coding. We best possibly mitigated this potential source of bias by requiring multiple diagnoses over an extended period of time.

We only had cost data for rehabilitation if the statutory health insurance was the third party payer, this was the case in most of the rehabilitations (GOLD Grade 1 = 67.7%, GOLD Grade 2 = 67.3%, GOLD Grade 3 = 68.4%, and GOLD Grade 4 = 73.9%). Interestingly, the length of rehabilitation stay differs tremendously according to the payer, so the mean is 23.4 days if the health insurance, and 27.8 days if the pension fund, is the third party payer. This adds up to a potential underestimation of approximately 30 to 40% of the reported rehabilitation costs in our analysis.

Direct health care costs were estimated form the perspective of a third-party payer, while the perspective for indirect costs were a societal. This methodological inconsistency is caused by the data basis. Direct costs would likely have been higher in a societal perspective, too, as we did not consider aspects such as out-of-pocket payments or support provided by family members.

Also the calculation of indirect costs with claims data is associated with some methodological difficulties. Premature retirement is based on insurance status of the patients, which is not further specified, therefore we were not aware of the real reason of premature retirement. Further, the costs of incapacity for work are underestimated in our results for two reasons. Firstly, a sickness notification issued by a physician has only be presented the employer for three or more days. Secondly, not all sickness notifications are handed in to the statutory health insurance, as only incapacity for work longer than 6 weeks is relevant to disbursement of sickness benefit. Thirdly, we had to use mean annual labor costs instead of the patient specific annual income, as for premature retired persons we only had actual retirement earnings, which are way lower than the potential earnings on the job market. Fourthly, in our analysis we used the human capital approach, which might overestimate the costs, instead of the friction costs approach. However, the discussion about the most appropriate approach is still on-going [48–52], and it seems that the friction cost approach allows more realistic estimates of productivity costs. Nevertheless, for conducting the friction cost approach in a sound manner, the professions (specialty and quality) of each person is necessary, to estimate the period a worker could be replaced completely, which unfortunately is inadequately mapped in claims data. Fifthly, we did not consider costs for presentism since we had no means of corresponding operationalization. In a Dutch study conducted by van Boven et al. [53] for a working COPD population aged between 45 and 65 years, the impairment of work, including absenteeism and presenteeism, found annual costs in the COPD population of €63.1 million, where presenteeism account for more than 66% of impaired work [21]. Therefore, not considering presenteeism leads to an underestimation of indirect costs about twice the costs for sickness leave.

In our analyses we used yearly income, as information on education are not available in claims data, as a control variable for socioeconomic status. This might be problematic as yearly income correlates with insurance status (e.g. working or retired) which is directly associated with indirect costs.

Despite these limitations in data and methodology, we are strongly convinced that a claims data-based approach is well suited to provide comprehensive insights into current costs structures of patients enrolled in a structured DMP for COPD. We analyzed a large unselected data set, with almost 40,000 COPD patients, which allowed us a stratification for GOLD grades while remaining with a sound sample size for the single strata. Besides claims data of a statutory health insurance fund we also used DMP COPD documentation data including clinical measures such as FEV_1_, weight and height allowing a FEV_1_ percentage prediction to classify the patients according to GOLD grades. The biggest strength is, besides the unselected patient population, that our direct cost calculations are based on real expenditures of a large regional statutory health insurance fund and not on weighted average costs as used in questionnaire based observational cohort studies, which also prone to recall bias.

## Conclusion

In conclusion, our results, based on a large unselected real-world dataset of patients enrolled in a structured DMP for COPD, demonstrate that more severe GOLD grades are associated with substantial additional direct and indirect costs, and with comorbidities independently significantly increase costs. In direct cost categories hospitalization was the main cost driver and in indirect costs premature retirement. All in all, COPD causes tremendous costs for third party payers, but even more for society as indirect costs exceed direct costs almost 2 times in grade 3 and 4 and more than 3 times in grade 1 and 2. Based on the results of international cost-of-illness studies, there is a continuous annual increase in average costs per COPD patient in each GOLD grade. From a statutory health insurance fund perspective as well as from a societal perspective prevention and interventions focusing on a better disease control, to avoid hospitalizations, and slowing down disease progression and strengthening the ability to work would be beneficial not only in terms of patient-relevant endpoints such as survival and health-related quality of life, but they also carry the potential to reduce costs associated with COPD.

## Supplementary information

**Additional file 1: Table S1.** Characteristics included vs. excluded patients. **Table S2.** Unadjusted healthcare utilization, work absenteeism and resulting costs included vs. excluded patients. (DOCX 22 kb)

## Data Availability

The datasets generated and/or analysed during the current study are not publicly available due §75 SGB X to but are available from the corresponding author on reasonable request.

## Abbreviations

ANOVA: Analysis of variance
BMI: Body mass index
COPD: Chronic obstructive pulmonary disease
COSYCONET: German COPD and Systematic Consequences – Comorbidities Network
DMP: Disease Management Program
DRG: Diagnoses Related Groups
EBM: (Einheitlicher Bewertungsmaßstab)
FEV_1_: Forced expiratory volume in 1 s
FVC: Forced vital capacity
GLM: Generalized linear model
GOLD: Global Initiative for Chronic Obstructive Lung Disease
LQ-DMP: Lebensqualität im Disease Management Programm COPD
OECD: Organisation for Economic Co-operation and Development
