# Supplementary material for: Direct and indirect costs of COPD progression and its comorbidities in a structured disease management program: results from the LQ-DMP study
Source: Respir Res. 2019 Oct 10;20:215. doi: 10.1186/s12931-019-1179-7 (PMC6785905; doi:10.1186/s12931-019-1179-7)
Supplement: Supplementary file 1 — Additional file 1: Table S1. Characteristics included vs. excluded patients. Table S2. Unadjusted healthcare utilization, work absenteeism and resulting costs included vs. excluded patients. (DOCX 22 kb) [file 12931_2019_1179_MOESM1_ESM.docx]

**Additional file 1: Table S1. Characteristics included vs. excluded patients**

|  | **Included** | **Excluded** | **p-value** |
| --- | --- | --- | --- |
| **N** | 39,307 (77.4%) | 11,494 (22.6%) |  |
| **Age (years)** | 70.0 (10.9) | 67.9 (11.9) | <0.0001 |
| **Age > 74 years** | 14,905 (37.9%) | 3,701 (32.20%) | <0.0001 |
| **Age 65-74 years** | 12,432 (31.6%) | 53,406 (29.6%) |  |
| **Age 55-64 years** | 8,698 (22.1%) | 2,817 (24.5%) |  |
| **Age <55** | 3,272 (8.3%) | 1,570 (13.7%) |  |
| **Female** | 17,865 (45.5%) | 5,366 (46.7%) | P=0.0194 |
| **income < €5.000** | 9,119 (23.2%) | 3,236 (28.2%) | <0.0001 |
| **income€5,000 < €10,000** | 13,513 (34.4%) | 3,668 (31.9%) |  |
| **income €10,000 <€15,000** | 8,282 (21.1%) | 1,881 (16.4%) |  |
| **income €15,000 < €20,000** | 3,017 (7.7%) | 768 (6.7%) |  |
| **income €20,000 < €30,000** | 3,012 (7.7%) | 941 (8.2%) |  |
| **income €30,000 < €50,000** | 2,041 (5.2%) | 866 (7.5%) |  |
| **income ≥ €50.000** | 323 (0.8%) | 134 (1.2%) |  |
| **Current smokers** | 12,196 (31.0%) | 4,352 (37.9%) | <0.0001 |
| **Former smokers (quit within the last 8 years)** | 5,523 (14.1%) | 630 (5.5%) |  |
| **Never smokers or former smokers (quit > 8 years ago)** | 21,588 (54.9%) | 6,512 (56.7%) |  |
| **Diabetes** | 14,030 (35.7%) | 3,678 (32.0%) | <0.0001 |
| **Stroke** | 11,414 (29.0%) | 2,707 (23.6%) | <0.0001 |
| **Myocardial infarction** | 5,114 (13.0%) | 1,281 (11.1%) | <0.0001 |
| **Cancer** | 12,108 (30.8%) | 2,799 (24.4%) | <0.0001 |
| **Arthritis** | 5,248 (13.4%) | 1,215 (10.6%) | <0.0001 |
|  |  |  |  |
| **N** | 39,307 (77.6%) | 11,318 (22.4%) |  |
| **BMI (kg/m²)** | 28.87 (6.1) | 28.75 (6.2) | P=0.0629 |
| **Normal weight (18.5 ≤ BMI < 25)** | 9,756 (24.8%) | 2,903 (25.7%) | P<0.05 |
| **Overweight (25 ≤ BMI < 30)** | 13,859 (35.3%) | 4,080 (36.1%) |  |
| **Obese (BMI ≥ 30)** | 14,886 (37.9%) | 4,098 (36.2%) |  |
| **Underweight (BMI < 18.5)** | 806 (2.1%) | 237 (2.1%) |  |

**Additional file 1: Table S2. Unadjusted healthcare utilization, work absenteeism and resulting costs included vs. excluded patients**

|  | **Included** | **Excluded** | **p-value** |
| --- | --- | --- | --- |
| N | 39,307 (78.8%) | 10,568 (21.2%) |  |
| **Healthcare utilization** | | | |
| **Outpatient services** | | | |
| % User | 100.0 | 99.9 | P<0.0001 |
| Total number of visits | 15.7 (8.8) | 15.1 (8.9) | P<0.0001 |
| General practitioner | 8.3 (5.8) | 7.7 (5.8) | P<0.0001 |
| Specialist | 6.3 (5.4) | 5.7 (5.2) | P<0.0001 |
| **Inpatient services** | | | |
| % User | 13.5 | 14.6 | P<0.001 |
| Number of hospital days | 5.2 (13.3) | 6.0 (14.6) | P<0.0001 |
| **Prescribed medication** | | | |
| %User | 98.2 | 98.0 | P=0.2489 |
| Number of prescribed drugs | 17.3 (13.1) | 17.6 (14.1) | P=0.0128 |
|  | | | |
| **Direct costs** | | | |
| Outpatient costs | €583 (1,456) | €545 (1,563) | P<0.05 |
| Inpatients costs | €2,429 (6,310) | €2,761 (8,872) | P<0.0001 |
| Medication costs | €753 (2,444) | €794 (2,573) | P=0.1275 |
| Rehabilitation | €151 (790) | €148 (840) | P=0.7647 |
| Aids and Remedies | €480 (1,096) | €531 (1,492) | P<0.001 |
| Travel costs | €147 (551) | €157 (561) | P=0.0858 |
| Total direct costs | €4,542 (8,139) | €4,936 (10,598) | P<0.0001 |
|  | | | |
|  | **Included** | **Excluded** | **p-value** |
| N | 11,970 (74.9%) | 4,018 (25.1%) |  |
| **Works absenteeism (participants <65 years)** | | | |
| %retired | 27.90 | 23.8 | P<0.0001 |
| %employed | 46.2 | 53.8 | P=0.8533 |
| % with sick days | 79.8 | 81.4 | P=0.1358 |
| Number of sick days | 46.1 (70.1) | 48.14 (73.0) | P=0.2913 |
| **Indirect costs (participants < 65 years)** | | | |
| Sick days | €3,100 (6,800) | €3,386 (7,207) | P<0.05 |
| Premature retirement | €11,102 (18,004) | €9,417 (17,041) | P<0.0001 |
| Total indirect costs | €14,202 (17,736) | €12,803 (17,045) | P<0.0001 |
